# Correlation Between Fecal Metabolomics and Gut Microbiota in Obesity and Polycystic Ovary Syndrome

**DOI:** 10.3389/fendo.2020.00628

**Published:** 2020-09-08

**Authors:** Ling Zhou, Zhexin Ni, Jin Yu, Wen Cheng, Zailong Cai, Chaoqin Yu

**Affiliations:** ^1^Department of Gynecology of Traditional Chinese Medicine, Changhai Hospital, Naval Medical University, Shanghai, China; ^2^Department of Biochemistry and Molecular Biology, Naval Medical University, Shanghai, China

**Keywords:** polycystic ovary syndrome, obesity, untargeted metabolomics, gut microbiota, biomarkers

## Abstract

**Objective:** This study aimed to explore the relationship between the fecal metabolites and gut microbiota in obese patients with PCOS and provide a new strategy to elucidate the pathological mechanism of obesity and PCOS.

**Methods:** The fecal samples of obese patients with PCOS (*n* = 18) and obese women without PCOS (*n* = 15) were analyzed by 16S rRNA gene sequencing and untargeted metabolomics. The peripheral venous blood of all subjects was collected to detect serum sex hormones. The association among fecal metabolites, gut microbiota, and serum sex hormones was analyzed with the R language.

**Results:** A total of 122 named differential fecal metabolites and 18 enrichment KEGG pathways were obtained between the groups. Seven fecal metabolites can be used as characteristic metabolites, including DHEA sulfate. The richness and diversity of gut microbiota in the obese PCOS group were lower than those in the control group. *Lachnoclostridium, Fusobacterium, Coprococcus_2*, and *Tyzzerela 4* were the characteristic genera of the obese patients with PCOS. Serum T level significantly and positively correlated with the abundance of fecal DHEA sulfate (*p* < 0.05), and serum DHEAS level significantly and negatively correlated with the abundance of fecal teasterone (*p* < 0.05).

**Conclusion:** Specific fecal metabolites may be used as characteristic metabolites for obese patients with PCOS. The closely relationship among gut microbiota, fecal metabolites, and serum sex hormones may play a role in the related changes caused by hyperandrogenemia.

## Introduction

Polycystic ovary syndrome (PCOS) is a chronic disorder of ovulation caused by abnormal reproductive endocrine and metabolic functions. The incidence rate of PCOS in women of child-bearing age is 9–18%, and its etiology remains unclear (1, 2). Although obesity is not included in the diagnosis standard of PCOS, according to the diagnostic criteria for obesity in China (3), ~38–88% of patients with PCOS are overweight or obese. The incidence rate of PCOS in obese people is much higher than that in people with normal weight (4). In patients with PCOS, obesity aggravates their reproductive dysfunction, further affects their pregnancy outcomes, and improves their risk of reproductive system tumors and cardiovascular diseases (5). At present, no breakthrough exists in the treatment of obesity (6).

Gut microbiota is an indispensable “microbial organ” of the human body that affects hosts' material metabolism and immune function and plays an important role in endocrine and metabolic diseases, such as obesity, type 2 diabetes, asthma, and autoimmune diseases (7). The incidence of PCOS is closely related to gut microbiota. Guo et al. (8) found that compared with a control group, PCOS rats have abnormal estrous cycles, increased androgen synthesis, polycystic ovarian changes, and decreased ovarian granulosa cell layers. Furthermore, the intestines of these rats demonstrate decreased abundance of *Lactobacillus, Ruminococcus*, and *Clostridium* and increased abundance of *Prevotella*. A prospective study by Lindheim et al. (9) including 24 patients with PCOS and 19 healthy women showed that endotoxemia caused by gastrointestinal leakage is associated with chronic inflammation, insulin resistance (IR), fat accumulation, and hyperandrogenism. Zhang et al. (10) further confirmed that IR and menstrual disorders in patients with PCOS are closely related to increased intestinal permeability. Our previous study (11) showed that the richness and diversity of gut microbiota in patients with PCOS are lower than those in healthy women. The characteristic gut microbiota of obese patients with PCOS and non-obese patients with PCOS is *Coprococcus_2* and *Lactococcus*, respectively. The different bacterial genera are likely to affect the intestinal environment of hosts through different intestinal enrichment and metabolism patterns and influence the occurrence and development of PCOS with and without obesity. However, the function of the different genera and their effects on the pathological mechanism of PCOS remain unclear.

Metabolomics seeks for the relative relationship among specific metabolites, a disease, and its phenotypic changes through qualitative and quantitative analysis of blood, urine, feces, and other body fluids. Most of the research objects are small molecules with a relative molecular mass of <1,000 (12). Fecal metabolites can reflect the status of gut microbiota and the relationship between symbiotic flora and host. The combination of fecal metabolomics and 16S rRNA gene sequencing is helpful to explain the close relationship between gut microbiota and host. Wen et al. (13) found that compared with a control group, a mouse model of chronic migraine shows significantly decreased and increased abundance of Firmicutes and Bacteroides, respectively; furthermore 14 metabolites, such as lysine, omega-muricholate, and glutamic acid, were screened as potential biomarkers for the mouse model by using 16S rRNA gene sequencing and fecal metabolomics. Bárcena et al. (14) also used the two methods and found that *Akkermansia* abundance positively correlated with the level of secondary bile acids and that the recovery of secondary bile acid metabolism or the reconstruction of gut microbiota is conducive to the treatment of premature aging syndrome.

In the current study, we determined the characteristic gut microbiota and their metabolites in obese patients with PCOS and further explored the correlation among fecal metabolites, gut microbiota, and serum hormones. The possible mechanism of obesity and PCOS should be revealed from a new perspective.

## Materials and Methods

### Participants

From June 2016 to November 2017, obese patients with PCOS (PCOS group, *n*=18) were recruited in the Traditional Chinese Medicine Gynecology Clinic of Shanghai Changhai Hospital. The inclusion criteria were based on the PCOS diagnostic criteria revised by the Rotterdam conference in the Netherlands in 2003, an age of 16–35 years and a BMI of ≥ 28 kg/m^2^ (3). The exclusion criteria included the use of oral contraceptives, antiandrogens, or insulin sensitizers in the past 3 months; pregnancy; other known causes of hyperandrogenism and ovulation disorders, such as 21-hydroxylase deficiency, congenital adrenal hyperplasia, Cushing's syndrome, androgen secreting tumor, thyroid disease, and hyperprolactinemia; any mental or organic diseases; the use of corticosteroids or sex steroids; past 2 years of drug use and alcohol abuse; and the use of antibiotics or probiotics or prebiotics in the past 3 months. Obese women without PCOS were enrolled as the control group (*n* = 15). Their age and BMI were matched with the patients, and they all had normal menstrual cycles, excluding the volunteers with clinical and/or biochemical hyperandrogenemia, any congenital disease or mental disease, and the abovementioned exclusion criteria of the PCOS group. This study has been approved by China Ethics Committee of Registering Clinical Trials (No. CHiECRCT-20160050), and each subject voluntarily signed an informed consent before the study.

### Collection of Peripheral Venous Blood and Fecal Samples

On the third day of menstrual cycle, peripheral venous blood was collected from the subjects, and the levels of sex hormones including luteinizing hormone (LH), follicle stimulating hormone (FSH), estradiol (E2), testoserone (T), dehydroepiandrosterone sulfate (DHEAS) and prolactin (PRL) were detected immediately by chemiluminescence using a UniCel Dxl 800 Access Immunoassay System (Beckman Coulter, Brea, United States) in laboratory diagnostics department of the hospital. Fecal samples were obtained from participants 3–5 days after menstruation. The participants were guided with a carbohydrate-based diet (300 g/day) 3 days before sampling. We unified the sampling and sample treatment procedures for each participant to minimize the impact of non-sample factors on gut flora and metabolites. A sterile plastic spoon and tube were used to collect approximately 10 g of fresh fecal sample from each subject. The samples were placed in an ice box, transported to the laboratory within 2 h, and then stored at −80°C. The fecal metabolites in the two groups were analyzed by untargeted metabolomics. The structural characteristics of gut microbiota in the two groups were analyzed by 16S rRNA gene sequencing. Data were analyzed on the free online Majorbio cloud platform (www.majorbio.com).

### Analysis of Untargeted Metabolomics

For each group, six frozen stool samples (~60 mg) were randomly selected and separately packed with steel beads pre-cooled at −80°C. Metabolites in the samples were extracted using 400 μL of a methanol:water (4:1, v/v) solution. The mixture was allowed to settle at −20°C, treated by a high-throughput tissue crusher (Wonbio-96c, Shanghai Wanbo Biotechnology Co., LTD.) at 50 Hz for 6 min, vortex mixed for 30 s, and ultrasonicated at 40 kHz for 30 min at 5°C. The samples were placed at −20°C for 30 min to precipitate proteins. After centrifugation at 13,000 × g at 4°C for 15 min, supernatants were carefully transferred into sample vials for LC-MS analysis. Quality control sample by mixing equal volumes of all samples was injected at regular intervals (every six samples) throughout the analytical run to provide a set of data from which repeatability can be assessed.

LC-MS was performed on a Waters UPLC I-class system equipped with a binary solvent delivery manager and a sample manager coupled with a Waters VION IMS Q-TOF mass spectrometer equipped with an electrospray interface (Waters Corporation, Milford, USA). The mass spectrometric data were collected using a Waters VION IMS Q-TOF mass spectrometer equipped with an electrospray ionization source operating in either positive or negative ion mode. Source and desolvation temperatures were set to 120 and 500°C, respectively, with a desolvation gas flow of 900 L/h. Centroid data were collected from 50 to 1000 *m*/*z* at a scan time of 0.1 s and an interscan delay of 0.02 s over a 13 min analysis time. Before pattern recognition, the original data were subjected to data preprocessing via baseline filtering, peak identification, integration, retention time correction, peak alignment, and normalization by using the instrument's own metabolomics processing software Progenesis QI (Waters Corporation, Milford, USA). Finally, a data matrix of retention time, mass-to-charge ratio, and peak intensity were obtained.

Positive and negative data were combined to obtain a combined data set, which was imported into the SIMCA-P+ 14.0 software package (Umetrics, Umeå, Sweden). Principal component analysis (PCA) and orthogonal partial least squares-discriminant analysis (OPLS-DA) were carried out to visualize metabolic alterations among the experimental groups after mean centering and unit variance scaling. Variable importance in projection (VIP) ranks the overall contribution of each variable to the OPLS-DA model. Variables with VIP > 1.0 and *p* < 0.05 were considered relevant for group discrimination. In this study, default 7-round cross-validation was applied with 1/7th of the samples being excluded from the mathematical model in each round to guard against overfitting. Finally, the differential metabolites were annotated through the metabolic pathways in the KEGG database (https://www.kegg.jp/kegg/pathway.html) to obtain the pathways involved in the differential metabolites. Pathway enrichment analysis was performed through the Python software package scipy.stats (https://docs.scipy.org/doc/scipy/), and the biological pathway most relevant to the experimental treatment was obtained through Fisher's exact test.

### 16S rRNA Gene Sequencing

The DNA extraction and PCR amplification of the fecal samples and the Illumina MiSeq sequencing and preprocessing of data have been described in our previous studies (9). An OTU abundance table was used to analyze the intestinal microbial diversity and abundance of the two groups. The community composition of each sample at different classification levels was determined. The alpha diversity indices, including Shannon, Sobs, and Ace, of the two groups were analyzed using the Mothur software (version v.1.30.1). The R language was used to draw a bar map of horizontal community at the phylum level. Beta diversity was analyzed using principal co-ordinate analysis (PCoA). Wilcoxon rank sum test was used to analyze for differential species between the two groups. Linear discriminant analysis (LDA) was used to estimate the influence of the abundance of each species on the differences between groups and form an LDA table. Correlation between fecal metabolites and gut microbiota was analyzed with the R language (pheatmap package). Pearson correlation coefficient was used to express the correlation degree between them. The correlation coefficient was displayed in the form of a thermogram, and the correlation between fecal metabolites and gut microbiota was reflected by a color gradient.

### Statistical Analysis

Quantitative demographic and clinical data with normal distribution in the two groups were expressed as mean ± standard deviation and analyzed by Student's *t*-test. The quantitative sequencing data with non-normal distribution were analyzed by Wilcoxon rank sum test, and *p*-values were checked using Benjamini and Hochberg false discovery rate (FDR) (15, 16). The SPSS software (version 21.0) was used for analysis. A double-tailed *p* of < 0.05 indicates statistically significant difference.

## Results

### Comparison of Baseline Data Between the Two Groups

All of the subjects were Chinese Han women from Shanghai who had similar eating habits and received 3 days of standard diet guidance before the study. No significant difference was found in age and BMI between the obese patients with PCOS and controls, which minimized the influence of confounding factors on the experimental results. The serum LH level and LH/FSH ratio in the obese PCOS group were significantly lower than those in the control group (*p* < 0.001, *p* = 0.007). However, the serum E2, DHEAS, and T level were significantly higher than those in the control group (*p* < 0.001, *p* < 0.001, *p* < 0.001; Table 1).

**Table 1:** Characters of serum sex hormone levels between two groups.

**Sex hormone**	**Control group (*n* = 15)**	**Obese PCOS group (*n* = 18)**	***p*-value**
Age (years)	24 ± 1	26 ± 4	0.106
BMI (kg/m^2^)	29.72 ± 1.48	29.78 ± 1.40	0.895
LH (IU/L)	11.46 ± 1.87	8.34 ± 1.57	<0.001
FSH (IU/L)	6.73 ± 1.12	6.4 ± 1.56	0.496
LH/FSH	1.72 ± 0.25	1.39 ± 0.38	0.007
E2 (pg/ml)	68 ± 5	77 ± 6	<0.001
DHEAS (μg/dL)	213.6 ± 8.8	359 ± 45.2	<0.001
PRL (μg/L)	14.2 ± 3.2	15.85 ± 9.67	0.542
T (μg/L)	0.46 ± 0.12	0.84 ± 0.18	<0.001

*BMI, body mass index; LH, luteinizing hormone; FSH, follicle-stimulating hormone; E2, estradiol; DHEAS, dehydroepiandrosterone sulfate; PRL, prolactin; T, testosterone; PCOS, polycystic ovary syndrome*.

### General Characteristics of Fecal Metabolites in the Two Groups

**Figure 1:**
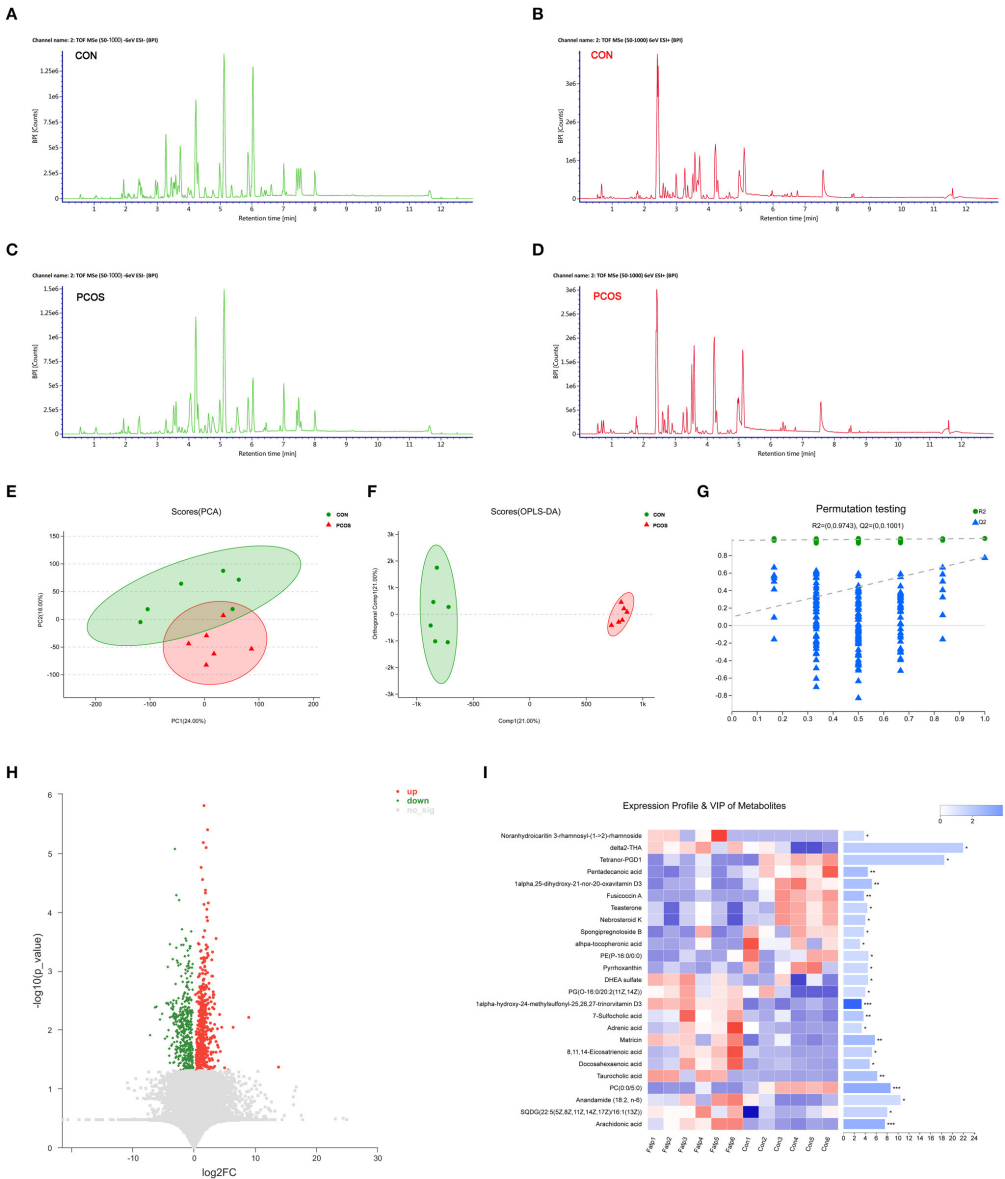
The difference of fecal metabolites between the two groups. Based peak ion flow chromatogram of fecal supernatant under negative **(A)** and positive ion mode **(B)** in control group, and under negative **(C)** and positive ion mode **(D)** in obese PCOS group. **(E)** PCA map. The distance of each coordinate point represents the degree of aggregation and dispersion between samples. **(F)** OPLS-DA map. The first prediction of Comp1 is mainly the decomposition degree, and the first orthogonality of orthogonal Comp1 is the decomposition degree. **(G)** Model verification map of OPLS-DA. The abscissa represents the replacement retention of the replacement test; the ordinate represents the R2 (green dot) and Q2 (blue triangle) replacement test values; and the two dashes represent the regression lines of R2 and Q2 respectively. **(H)** Volcanic map of differential metabolites. The abscissa is the multiple change value of the expression difference of metabolites between the two groups, and the ordinate is the statistical test value of the expression difference of metabolites, that is, *p*-value. The abscissa and ordinate values are all logarithmically processed. Each point in the figure represents a specific metabolite. **(I)** Heatmap of differential metabolites (VIP > 3, *p* < 0.05) between groups. The color represents the relative abundance of the metabolite in samples. On the right is the VIP bar graph of metabolites. The length of the bar represents the contribution value of the metabolite to the difference between the groups. *p < 0.05; **p < 0.01; ***p < 0.001.

A basic peak ion (BPI) flow diagram of fecal samples in the control group and obese PCOS group under the typical positive and negative ion mode is shown in Figures 1A–D. The baseline of BPI (±) flow graph of the two groups was stable, and the number and height of ion peaks were significantly different between the obese PCOS and control groups. This finding suggests that the detection results were reliable and that the fecal metabolites between the two groups showed significant differences. PCA (Figure 1E), OPLS-DA (Figure 1F), and model validation (Figure 1G) revealed significant differences between the distribution of samples, suggesting a significant difference of fecal metabolites between the two groups. A total of 948 differential metabolites (*p* < 0.05, VIP > 1, Figure 1H) were detected. Furthermore, 122 named differential metabolites were successfully quantified, including 25 metabolites with VIP > 3, such as arachidonic acid and taurocholic acid (Figure 1I).

### Differential Metabolites and KEGG Pathways

**Figure 2:**
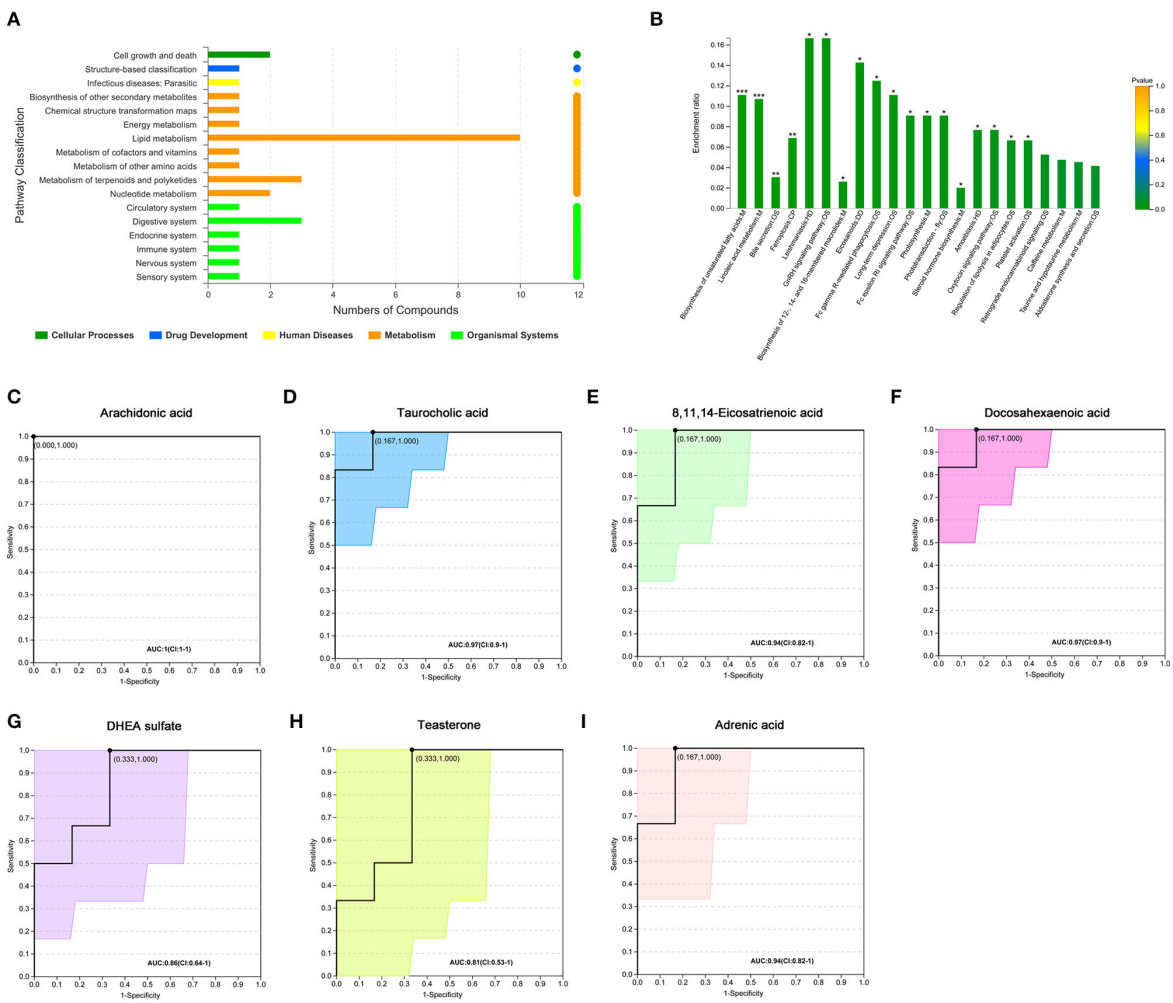
Analysis of KEGG pathway related to differential metabolites. **(A)** KEGG pathways on level 1 and level 2 related to differential metabolites. The ordinate is the name of pathway level 2, and the abscissa is the number of metabolites related to the pathway. Different colors represent different pathways on level 1. **(B)** KEGG pathway enrichment column chart. The abscissa is the name of pathway level 3. **(C–I)** Receiver operator characteristic curves of seven metabolites. Area under the curve (AUC) indicates the discrimination performance. Confidence interval (CI) represents 95% confidence interval of AUC calculated based on nonparametric resampling method. The point on the curve refers to the best threshold which is determined based on ROC curve to distinguish the two groups. The ordinate is the enrichment rate, that is, the ratio of the number of metabolites enriched in the pathway to the number of metabolites annotated in the pathway. *p < 0.05; **p < 0.01; ***p < 0.001.

We further analyzed 122 named differential metabolites on the KEGG pathways and found that most of the differential metabolites were involved in lipid metabolism (Figure 2A). A total of 17 differential metabolites were found to participate in pathway level 3 (Table 2), involving 22 specific pathways. The metabolites were significantly enriched in 18 KEGG pathways (*p* < 0.05). Biosynthesis of unsaturated fatty acids, linoleic acid metabolism, bile secretion, and ferroptosis were the four pathways with the most significant enrichment (*p* < 0.01, Figure 2B). We focused on seven metabolites with VIP > 3 and relative abundance > 1,000, including arachidonic acid (fold change, FC = 3.6), taurocholic acid (FC = 9.4), 8,11,14-eicosatrienoic acid (FC = 5.2), docosahexaenoic acid (FC = 4.8), DHEA sulfate (FC = 1.9), teasterone (FC = 0.66), and adrenic acid (FC = 3.7) (Table 2). These seven fecal metabolites were identified as characteristic metabolites for obese patients with PCOS.

**Table 2:** Relative abundance of seventeen significant differential metabolites involved in KEGG pathway analysis (Mean with SD).

**Metabolite**	**M/Z**	**Mode**	**PCOS**	**CON**	**VIP**	**FC**	***p*-value**	**State**
Arachidonic acid	303.2	neg	*5, 763*±*2, 170*	*1, 579*±529	7.6	3.6	0.001	up
Taurocholic acid	514.3	neg	*3, 408*±*2, 030*	364 ± 164	6.2	9.4	0.004	up
8,11,14-Eicosatrienoic acid	305.2	neg	*3, 082*±*2, 177*	595 ± 368	5.2	5.2	0.02	up
Docosahexaenoic acid	327.2	neg	*2, 456*±*1, 488*	512 ± 218	4.8	4.8	0.01	up
DHEA sulfate	367.2	neg	*3, 713*±*1, 115*	*1, 973*±*1, 297*	4.5	1.9	0.03	up
Teasterone	471.3	pos	*3, 478*±955	*5, 277*±*1, 333*	4.4	0.66	0.02	down
Adrenic acid	331.3	neg	*1, 539*±*1, 069*	421 ± 379	3.4	3.7	0.04	up
9,12,13-TriHOME	329.2	neg	843 ± 449	269 ± 252	2.5	3.1	0.02	up
plastoquinol-1	389.3	neg	350 ± 304	803 ± 320	2.2	0.44	0.03	down
Eicosapentaenoic acid	301.2	neg	389 ± 166	54 ± 27	2.1	7.2	0.0006	up
10-Deoxymethymycin	471.3	pos	80 ± 66	328 ± 182	1.7	0.24	0.01	down
Pregnenolone sulfate	395.2	neg	350 ± 120	181 ± 121	1.4	1.9	0.04	up
Xanthine	151.0	neg	81 ± 49	200 ± 58	1.3	0.41	0.003	down
Bilirubin	583.2	neg	137 ± 32	40 ± 27	1.2	3.5	0.0002	up
Nervonic acid	411.3	neg	19 ± 34	149 ± 121	1.1167	0.126	0.03	down
10-Deoxymethynolide	314.2	pos	123 ± 83	22 ± 24	1.0964	5.4708	0.02	up
Thymine	251.1	neg	32 ± 28	123 ± 78	1.0144	0.2606	0.02	down

*M/Z, mass-to-charge ratio; neg, negative ion scanning mode; pos, positive ion scanning mode; VIP, variable important in projection; FC, fold change; up, the expression of metabolite in obese PCOS group increased; down, the expression of metabolite in obese PCOS group decreased*.

### Characteristics of Gut Microbiota Between the Two Groups

**Figure 3:**
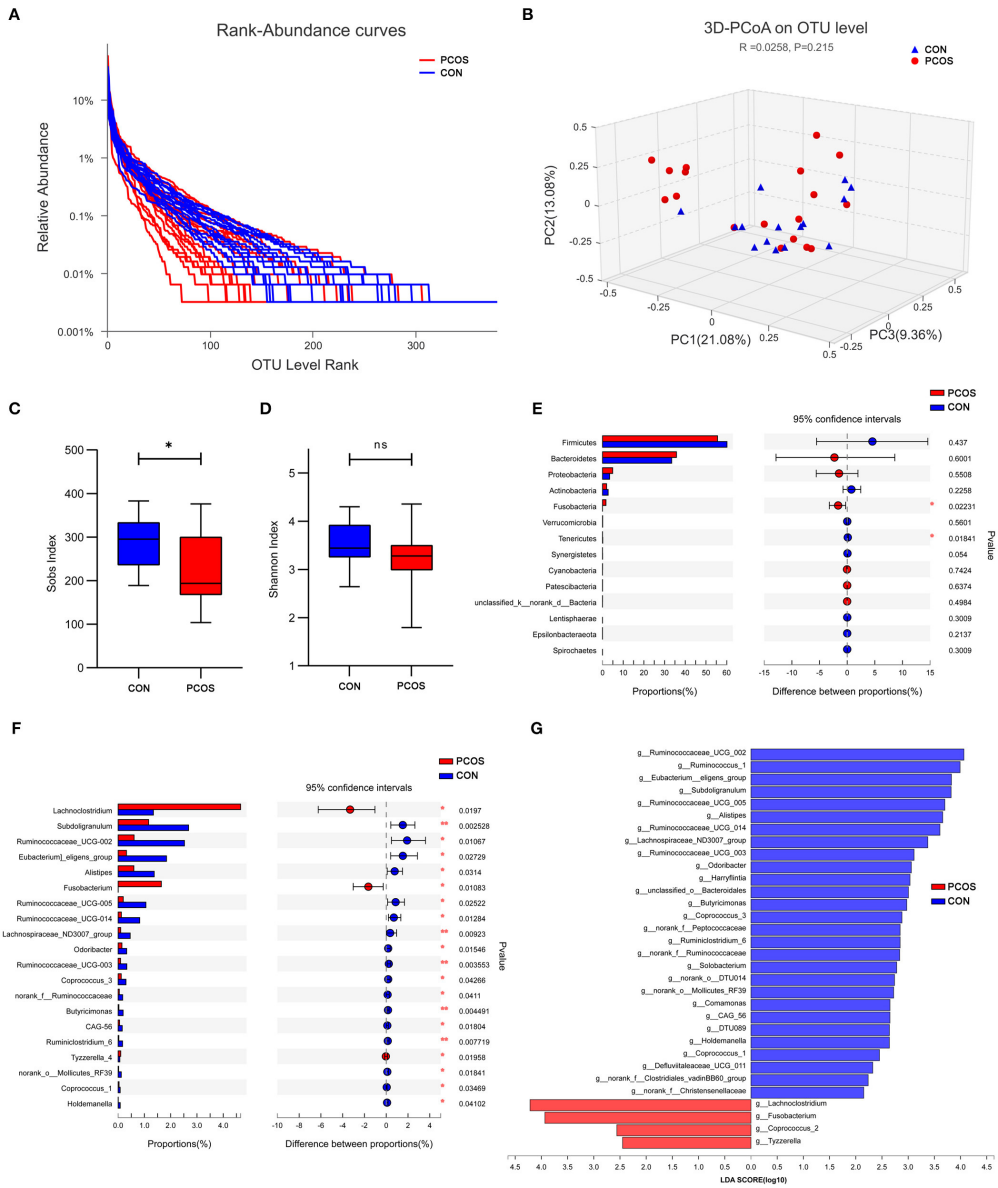
Characteristics of gut microbiota between the two groups. **(A)** Rank-Abundance curves. The abscissa represents the ranking level of OTU number, and the ordinate represents the relative percentage of OTU number. The abscissa position at the extension end of sample curve is the number of OTU of the sample. **(B)** 3D-PCoA chart. the X, Y and Z axis represent three selected principal axes, and the percentage represents the explanatory value of the principal axis to the difference of samples. **(C)** The Sobs index of obese PCOS group was significantly lower than that of control group, and **(D)** no significant difference on Shannon index was found between groups. **(E)** The column chart of species abundance on phylum level. **(F)** The column chart of top 20 species with significant difference on genus level. The left X-axis represents different groups, the Y-axis represents the average relative abundance of a species in different groups, and the right represents the confidence interval and *p*-value. **(G)** LDA chart. The score was obtained by LDA analysis (linear regression analysis). The greater the LDA score, the greater the impact of the representative species abundance on the differences between groups. *p < 0.05; **p < 0.01.

At the OTU classification level, the rank-bank curve of the obese PCOS group decreased more sharply than that of the control group, suggesting that gut microbiota diversity was decreased in the PCOS group (Figure 3A). PCoA showed no significant difference between the two groups (Figure 3B). Compared with the control group, the obese PCOS group showed significantly lower Sobs index (*p* < 0.05, Figure 3C) and lower Shannon index with no statistically significant difference (*p* > 0.05, Figure 3D). These results suggest that the richness and diversity of gut microbiota in the obese PCOS group were lower than those of the control group. At the phylum level, the obese PCOS group showed decreased ratio of Firmicutes/Bacteroides (1.98 vs. 1.57), significantly increased abundance of Fusobacteria (*p* = 0.022), and significantly decreased abundance *of* Tenericutes (*p* = 0.018, Figure 3E, Table 3). Among the top 20 genera with significant difference, *Lachnoclostridium, Fusobacterium*, and *Tyzzerella_4* showed significantly higher abundance in the obese PCOS group compared with the control group; the other 17 genera were significantly lower in abundance in the obese PCOS group compared with the control group (Figure 3F). LDA showed that *Lachnoclostridium, Fusobacterium, Coprococcus_2*, and *Tyzzerella* were the important characteristic genera in the obese PCOS group compared with the control group (Figure 3G).

**Table 3:** The relative abundances of species on phylum and genus level.

	**Control (*****n*** **=** **15)**		**Obese PCOS (*****n*** **=** **18)**		
**Species on phylum level**	**Median**	**IQR (1/4, 3/4)**	**Median**	**IQR (1/4, 3/4)**	***p-*value**
*Firmicutes*	0.6333	0.1952 (0.5024, 0.6976)	0.5858	0.2216 (0.4499, 0.6715)	0.437
*Bacteroidetes*	0.2988	0.2352 (0.2134, 0.4486)	0.3445	0.2548 (0.2442, 0.499)	0.600
*Proteobacteria*	0.0173	0.0145 (0.0133, 0.0278)	0.0235	0.0577 (0.0115, 0.0692)	0.551
*Actinobacteria*	0.0219	0.0401 (0.0061, 0.0462)	0.0067	0.0327 (0.0023, 0.035)	0.226
*Fusobacteria*	0.0000	0.0001 (0, 0.0001)	0.0001	0.008 (0, 0.008)	0.022
*Verrucomicrobia*	0.0000	0.0006 (0, 0.0006)	0.0000	0.0001 (0, 0.0001)	0.560
*Tenericutes*	0.0002	0.0015 (0, 0.0015)	0.0000	0 (0, 0)	0.018
**Species on genus level**	**Median**	**IQR (1/4, 3/4)**	**Median**	**IQR (1/4, 3/4)**	***p*****-value**
& *Lachnoclostridium*	0.0099	0.0125 (0.0067, 0.0192)	0.0213	0.051 (0.0119, 0.0629)	0.020
& *Subdoligranulum*	0.0214	0.0226 (0.0136, 0.0362)	0.0045	0.0151 (0, 0.0151)	0.003
& *Ruminococcaceae_UCG-002*	0.0163	0.0362 (0.0025, 0.0387)	0.0005	0.0086 (0, 0.0086)	0.011
& *Eubacterium]_eligens_group*	0.0064	0.0251 (0.0008, 0.0259)	0.0011	0.0055 (0, 0.0055)	0.027
# *Alistipes*	0.0121	0.019 (0.0015, 0.0205)	0.0016	0.0095 (0.0001, 0.0096)	0.031
♦*Fusobacterium*	0.0000	0 (0, 0)	0.0001	0.008 (0, 0.008)	0.011
& *Ruminococcaceae_UCG-005*	0.0040	0.0109 (0.0001, 0.011)	0.0000	0.0025 (0, 0.0025)	0.025
& *Ruminococcaceae_UCG-014*	0.0041	0.0114 (0, 0.0114)	0.0000	0 (0, 0)	0.013
& *Lachnospiraceae_ND3007_group*	0.0017	0.0019 (0.0009, 0.0028)	0.0002	0.0016 (0, 0.0016)	0.009
# *Odoribacter*	0.0025	0.0029 (0.0013, 0.0042)	0.0008	0.0013 (0, 0.0013)	0.015
& *Ruminococcaceae_UCG-003*	0.0023	0.0039 (0.001, 0.0049)	0.0005	0.0016 (0, 0.0016)	0.004
& *Coprococcus_3*	0.0021	0.0031 (0.0008, 0.0039)	0.0003	0.0021 (0, 0.0021)	0.043
& *norank_f__Ruminococcaceae*	0.0006	0.0018 (0, 0.0018)	0.0000	0.0002 (0, 0.0002)	0.041
# *Butyricimonas*	0.0012	0.0028 (0.0003, 0.0031)	0.0002	0.0005 (0, 0.0005)	0.004
& *CAG-56*	0.0009	0.0026 (0.0004, 0.003)	0.0000	0.0007 (0, 0.0007)	0.018
& *Ruminiclostridium_6*	0.0009	0.0026 (0, 0.0026)	0.0000	0 (0, 0)	0.008
& *Tyzzerella_4*	0.0000	0.0001 (0, 0.0001)	0.0001	0.0019 (0, 0.0019)	0.020
Δ *norank_o__Mollicutes_RF39*	0.0002	0.0015 (0, 0.0015)	0.0000	0 (0, 0)	0.018
& *Coprococcus_1*	0.0007	0.0012 (0.0002, 0.0014)	0.0000	0.0005 (0, 0.0005)	0.035
& *Holdemanella*	0.0000	0.0006 (0, 0.0006)	0.0000	0 (0, 0)	0.041

*The sum of all species richness at the same classification level of each sample is set as 1. Different symbols represent different phylum: &, Firmicutes; #, Bacteroidetes; ♦, Fusobacteria; Δ, Tenericutes*.

### Correlation Analysis of Fecal Metabolites With Gut Microbiota and Serum Sex Hormone in the Obese PCOS Group

**Figure 4:**
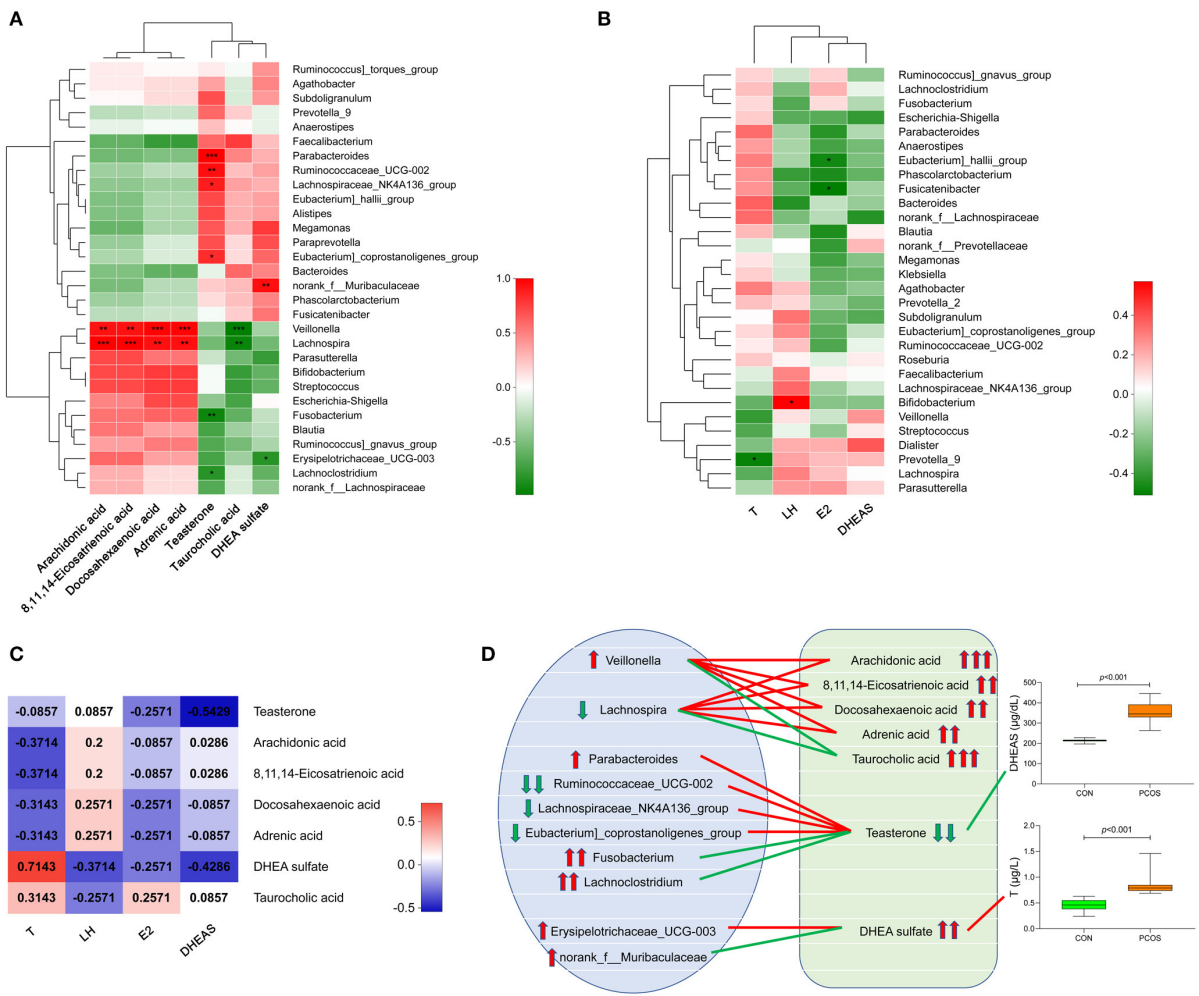
Correlation analysis of gut microbiota, fecal metabolites and serum sex hormones in obese PCOS group. **(A)** Correlation heatmap between seven important differential fecal metabolites and the top 30 genera in abundance. **(B)** Correlation heatmap between serum sex hormones and the top 30 genera in abundance. **(C)** Correlation heatmap between serum sex hormones and seven important differential fecal metabolites. Different colors represent correlation level; *p < 0.05; **p < 0.01, ***p < 0.001. **(D)** Correlation map among gut microbiota, fecal metabolites and serum sex hormones. The red line represents a significant positive correlation, while the green line represents a significant negative correlation; the up arrow represents an increase in abundance, while the down arrow represents a decrease in abundance; one, two and three arrows represents *p* > 0.05, *p* < 0.01, and *p* < 0.001, respectively.

We analyzed the correlation between the seven important differential metabolites and the top 30 genera in the obese PCOS group (Figure 4A). We found that the abundance of *Veillonella* and *Lachnospira* positively correlated with that of fecal arachidonic acid, 8,11,14-eicosatrienoic acid, and docosahexaenoic acid (*p* < 0.01) but negatively correlated with that of fecal taurocholic acid (*p* < 0.01). In addition, the abundance of fecal teasterone positively correlated with that of *Paracharacteroids* and *Ruminococcaceae_UCG-002* (*p* < 0.01) but negatively correlated (*p* < 0.01) with that of *Fusobacterium*. In the aspect of sex hormones (Figure 4B), serum T level negatively correlated with the abundance of *Prevotella_9* (*p* < 0.05), serum LH level positively correlated with the abundance of *Bifidobacterium* (*p* < 0.05), serum E2 level negatively correlated with the abundance of *Eubacterium)_hallii_group* and *Fusicatenibacter* (*p* < 0.05), and no bacterial group significantly correlated with serum DHEAS level. Furthermore, serum T level significantly and positively correlated with the abundance of fecal DHEA sulfate (*p* < 0.05); and serum DHEAS level significantly and negatively correlated with the abundance of fecal teasterone (*p* < 0.05, Figure 4C). On the basis of the relationship among fecal metabolites, gut microbiota and serum sex hormones, we drew a correlation diagram to show their connection intuitively (Figure 4D).

## Discussion

In this study, we revealed the differences in fecal metabolites, gut microbiota, and serum sex hormone levels between the obese patients with PCOS and control group and discussed the correlation among the three factors. On the basis of the analysis of differential fecal metabolites between the two groups, we screened out the important KEGG enrichment pathways. And we determined seven characteristic metabolites from fecal metabolites, which may be used to distinguish PCOS. The diversity and richness of gut microbiota in the obese PCOS group were significantly lower than those in the control group. Correlation analysis revealed a close association among the important differential metabolites, serum sex hormones, and the top 30 species of gut microbiota in the obese patients with PCOS.

Untargeted metabolomics allowed the detection of 122 named differential fecal metabolites, including arachidonic acid and taurocholic acid. The metabolites were classified as lipids and lipid-like molecules (75%), organoheterocyclic compounds (12.5%), organic compounds (6.25%), and organic oxygen compounds (6.25%).

Most obese PCOS patients present with abdominal obesity, whose fat accumulation and metabolic disorder will activate lipolytic enzymes, thereby accelerating fat catabolism, forming a large amount of free fatty acids, which in turn leads to insulin resistance. Lipid metabolism disorders may be related to abnormal levels of lipid metabolism-related regulatory factors, such as adiponectin, leptin, and endolipid. Adiponectin is a protein secreted by adipocytes, and its low level can increase the risk of cardiovascular disease (17). Leptin is a protein hormone synthesized and secreted by adipocytes; it can regulate the body's energy metabolism and reproductive function (18, 19). High leptin concentrations in the ovary can interfere with dominant follicle formation and oocyte maturation and increase the production of androgen in theca cells (18). Insulin can increase the metabolism of arachidonic acid through the cyclooxygenase-2 and cytochrome P450 epoxidase pathways, affecting the development and maturation of oocytes (20).

We further analyzed the KEGG pathways involved in the differential metabolites and found 18 significantly enriched KEGG pathways, including pathways for unsaturated fatty acids, linoleic acid metabolism, bile secretion, and ferroptosis. Excessive fatty acids affect liver glucose metabolism and inhibit the inactivation of insulin by the liver, resulting in increased blood insulin levels (21, 22). PCOS itself and hyperinsulinemia could increase the risk of gestational diabetes in women with PCOS by approximately three times compared with women without PCOS (23).

The current study have revealed seven differential metabolites that could be used as characteristic metabolites for obese PCOS patients, given their VIP > 3 and relative abundance > 1,000. DHEA is an active form transformed from DHEAS, and the latter is an important biomarker of adrenal androgen (24), which is highly correlated with male reproductive phenotype (25). In the current study, the fecal DHEAS level in the obese PCOS group was increased significantly, indicating that untargeted metabolomics was sufficiently sensitive for androgen detection.

Fecal metabolites are believed to have a close relationship with gut microbiota. Thus, we further analyzed the characteristics of gut microbiota in the two groups. A combination of alpha and beta diversity analysis showed a dysbacteriosis in the intestines of obese patients with PCOS, consistent with our previous study (11). Compared with the control group, the obese PCOS group showed significantly increased abundance of Fusobacteria and significantly decreased Firmicutes/Bacteroides ratio and abundance of Tenericutes. Firmicutes and Bacteroidetes are the two most abundant bacterial phyla in the human intestine, and disorders in their abundance are tightly related to obesity (26). In animal models, Bacteroides disorder induces chronic inflammation and colitis (27, 28). The increased abundance of Fusobacteria is a risk factor for obesity (29). We speculated that the increased abundance of Fusobacteria in the obese patients with PCOS was related to its characteristics in obesity. *Fusobacterium* is a flora belonging to Fusobacteria and settles normally in the human oral cavity, upper respiratory tract, gastrointestinal tract, and vaginal mucosa; its disorder can cause periodontitis, tonsillitis, and other diseases. McCoy et al. (30) confirmed that the abundance of *Fusobacterium* is increased significantly in the rectal mucosa of patients with adenoma. Kostic et al. (31) found that *Fusobacterium* is significantly enriched in the intestinal mucosa of patients with colorectal cancer. We speculate that *Fusobacterium* is related to the increased intestinal permeability of obese patients with PCOS, the specific mechanism of which requires further exploration. *Subdoligranulum* can inhibit intestinal inflammation and is decreased in obese patients with POCS; this bacterium is also closely related to chronic inflammation in these patients. Chronic non-specific inflammatory factors affect ovarian function, androgen synthesis in the body, and insulin resistance to affect follicular development, resulting in infertility and adverse pregnancy outcomes. In addition, the current study revealed that *Lachnoclostridium, Fusobacterium, Coprococcus_2*, and *Tyzzerella* are the four characteristic genera of obese patients with PCOS.

Correlation analysis revealed a linear relationship between gut microbiota, characteristic fecal metabolites, and serum sex hormones. However, which of these factors played a central role remains to be studied. We found that the abundance of *Veillonella* and *Lachnospira* in the obese patients with PCOS positively correlated with the abundance of fecal arachidonic acid, 8,11,14-eicosatrienoic acid, and docosahexaenoic acid and negatively correlated with the abundance of fecal taurocholic acid. Arachidonic acid is an essential n-6 long-chain polyunsaturated fatty acid that belongs to the ω-6 family of fatty acids. It is the precursor of prostaglandin and thromboxane A2, playing an important role in the esterification of cholesterol ester, inflammatory response, muscle growth, and platelet activation (32). PCOS has been proved to be a chronic inflammatory disease, and obesity and dyslipidemia are common symptoms. The arachidonic acid can be metabolized to leukotriene A4 (LTA4) and lipoxin (LXs), and further generate other types of leukotriene (LT) (33). LT can activate leukocytes and promote inflammation (34). Adipose tissue in obese PCOS patients can be activated by the inflammatory mediators and releases a large number of inflammatory factors. This series of biological process can inhibit insulin signal transduction and decrease the sensitivity of tissue cells to insulin, resulting in disorder of glycolipid metabolism and increase of blood glucose and lipids level, thus producing insulin resistance (35, 36). In addition, Khajeh et al. (37) uncovered that arachidonic acid has an important influence in oocyte maturation and embryo development by affecting the meiosis and ovulation of oocytes through the cAMP/PKC pathway. We speculate that the intestinal bacteria *Veillonella* and *Lachnospira*, through the metabolic pathway of arachidonic acid, could affect the blood glucose, lipid, and cholesterol concentration of obese women with PCOS.

Gut microbiota is not only affected by sex hormones but also has an impact on sex hormones (38). Intriguingly, Insenser et al. (39) revealed a closely relationship between gut microbiota and sex hormones in PCOS, which was also influenced by sex and obesity. However, they did not figure out linear associations between them and metabolic mechanism in intestine, which may help explain the puzzles in the disease. In the present study, we found that serum T level negatively correlated with the abundance of *Prevotella_9*, serum LH level positively correlated with the abundance of *Bifidobacterium*; and serum E2 level negatively correlated with the abundance of *Eubacterium]_hallii_group* and *Fusicatenibacter*. Human ovaries, adrenal glands, and adipose tissue can secrete sex hormones, enter the blood circulation in a free or combined state, and carry out hepatoenteron circulation in the liver and digestive tract. In the liver, free estrogens are converted into conjugated estrogens, which become water-soluble molecules and are excreted through urine or feces. β-Glucuronidase can dissociate conjugated estrogens (40). Free estrogens are re-absorbed, enter the blood circulation, and finally reach the target organs. Gut microbiota disorders can increase the content of β-glucuronidase in the intestinal tract, which can increase free estrogen levels in the host and cause a series of gynecological diseases (41). We speculate that *Bifidobacterium, Eubacterium]_hallii_group*, and *Fusicatenibacter* may be involved in the changes of estrogen metabolism in PCOS. We found a significant positive correlation between serum T level and the abundance of fecal DHEA sulfate in obese patients with PCOS and a significant negative correlation between serum DHEAS level and the abundance of fecal teasterone. Androgens in human body are produced in the adrenal cortex, gonads and peripheral tissues, and can be metabolized by gut microbiota (42, 43). DHEAS is a kind of androgen mainly secreted by adrenal cortex in PCOS women, and is converted from DHEA by the related enzymes. In adrenals, the main biosynthesis pathway of DHEAS production is pregnenolone and its subsequent hydroxylation to 17 hydroxypregnenolone after cholesterol cleavage, and then its C17 side chain breaks to form DHEA (44). Gut DHEAS may come from the excessive serum DHEAS and partly may be converted by the gut microbiota. Disordered gut microbiota and metabolites may act on multiple organs throughout the body by damaging the intestinal mucosal barrier, affecting the serum sex hormone levels, glycogen biosynthesis and metabolism, and causing systemic chronic inflammation (45, 46). DHEAS in the intestinal and peripheral circulation of obese patients with PCOS may be freely exchanged, whereas too much androgens can affect the diversity and richness of gut microbiota (47). Additionally, hyperandrogenism can increase the risk of type 2 diabetes in women, promote insulin resistance and visceral fat accumulation (48), which will aggravate the symptoms of obese PCOS patients. We believe that gut microbiota is involved in the physiological and pathological process of obese patients with PCOS and plays a role in the related changes caused by hyperandrogenemia.

This study has limitations. The number of subjects was relatively small, and the correlations were not verified. We will expand the sample size in future research and further verify the results in animal models. However, this study was the first to use fecal metabolomics combined with gut microbiota to explore the relationship between intestinal environment and clinical parameters in obese patients with PCOS. To some extent, the data provide strong support for the clinical diagnosis and treatment of obesity and PCOS.

In conclusion, untargeted metabolomics and 16S rRNA gene sequencing uncovered the characteristic changes of fecal metabolites and gut microbiota in obese patients with PCOS. Four characteristic intestinal species, including *Lachnoclostridium, Fusobacterium, Coprococcus_2*, and *Tyzzerela*, were observed in the patients. Seven fecal metabolites with VIP > 3 and relative abundance > 1,000, such as arachidonic acid, taurocholic acid, and DHEA sulfate, can be used as characteristic metabolites for obese patients with PCOS. In these patients, gut microbiota, fecal metabolites, and serum sex hormones were closely correlated, and the characteristic gut microbiota and their metabolites may play a role in the phenotypic changes caused by hyperandrogenemia.

## Data Availability Statement

The datasets presented in this study can be found in online repositories. The names of the repository/repositories and accession number(s) can be found below: NCBI SRA (accession: SRP262631).

## Ethics Statement

The studies involving human participants were reviewed and approved by China Ethics Committee of Registering Clinical Trials (No. CHiECRCT-20160050). The patients/participants provided their written informed consent to participate in this study.

## Author Contributions

CY and ZC conceived and supervised the study. LZ, ZN, JY, and WC collected and processed samples. LZ and ZN analyzed data and drafted the manuscript. All authors contributed to the article and approved the submitted version.

## Conflict of Interest

The authors declare that the research was conducted in the absence of any commercial or financial relationships that could be construed as a potential conflict of interest.
